# Advances in Engineering Cells for Cancer Immunotherapy

**DOI:** 10.7150/thno.38583

**Published:** 2019-10-16

**Authors:** Xiao Xu, Teng Li, Shiyang Shen, Jinqiang Wang, Peter Abdou, Zhen Gu, Ran Mo

**Affiliations:** 1 State Key Laboratory of Natural Medicines, Jiangsu Key Laboratory of Drug Discovery for Metabolic Diseases, Center of Advanced Pharmaceuticals and Biomaterials, China Pharmaceutical University, Nanjing 210009, China.; 2 Department of Bioengineering, University of California, Los Angeles, CA 90095, USA.; 3 Jonsson Comprehensive Cancer Center, California NanoSystems Institute, and Center for Minimally Invasive Therapeutics, University of California, Los Angeles, CA 90095, USA.

**Keywords:** Cell therapy, Drug delivery, Cancer immunotherapy, Cell engineering, Nanomedicine

## Abstract

Cancer immunotherapy aims to utilize the host immune system to kill cancer cells. Recent representative immunotherapies include T-cell transfer therapies, such as chimeric antigen receptor T cell therapy, antibody-based immunomodulator therapies, such as immune checkpoint blockade therapy, and cytokine therapies. Recently developed therapies leveraging engineered cells for immunotherapy against cancers have been reported to enhance antitumor efficacy while reducing side effects. Such therapies range from biologically, chemically and physically -engineered cells to bioinspired and biomimetic nanomedicines. In this review, advances of engineering cells for cancer immunotherapy are summarized, and prospects of this field are discussed.

## Introduction

Immunotherapies that either induce or inhibit the host immune response have been used in the clinical treatment of many diseases including infections 1-3, cancers 4, 5 and autoimmune diseases 6, 7. As opposed to cytotoxic drugs that directly kill pathogens or mutant cells, immunotherapeutics function by activating the patient's own immune system to eradicate the pathogens or mutant cells 8-10. The specific antigens produced by the pathogenic cells can be recognized and internalized by antigen-presenting cells (APCs), such as dendritic cells (DCs), which are subsequently presented on major histocompatibility complexes (MHCs) on the APC surfaces 11. When APCs with the MHC-bound antigens interact with T lymphocytes, the T lymphocytes become primed to recognize the antigens and attack the pathogenic cells 12-14. However, cancer cells often suppress host immune cells using various mechanisms in order to evade destruction and continue to proliferate 15-17.

Cancer immunotherapy, also referred to as immuno-oncology, aims to induce the immune system of host to identify and eliminate cancers 18-20. The primary cancer immunotherapeutic strategies currently being used in the clinic include cancer vaccines, immune checkpoint blockade (ICB) and adoptive cell transfer (ACT) therapy 21-23. Cancer vaccines are intended to enhance the autoimmune response against cancer cells, and are typically categorized into nucleic acid, viral or cellular vaccines 24-26. Nucleic acid vaccines contain DNA or RNA sequences that express specific proteins to activate APCs, which further activate T lymphocytes to promote anticancer activity 27. Virus vaccines act as viruses specifically proliferating in and killing cancer cells without harming normal cells. Oncolytic virus is clinically applied as an active drug for cancer therapy 28. Cell vaccines are engineered antigen-presenting cells that activate T cells to produce an immune response after entering into the body 29. Sipuleucel-T (Provenge^®^) is the first cell-based therapeutic vaccine approved by the U.S. Food and Drug Administration (FDA) to treat prostate cancer 30. The ICB therapy uses antibodies to block the immune-inhibitory interaction between cancer cells and immune cells in an effort to unlock the host antitumor response, and has demonstrated notable clinical outcomes 31-33. FDA-approved immune checkpoint inhibitors include ipilimumab 34, pembrolizumab 35, nivolumab 36, atezolizumab 37, avelumab 38, durvalumab 39 and cemiplimab 40. The ACT therapy utilizes the cytotoxic capabilities of T lymphocytes to kill cancer cells, including tumor-infiltrating lymphocyte (TIL) therapy 41, 42, T cell receptor (TCR)-engineered T (TCR-T) cell therapy 43, 44 and chimeric antigen receptor T (CAR-T) cell therapy 45, 46. The first success of ACT for cancer treatment witnessed the regression of melanoma treated with the *ex vivo* expanded TILs 47, 48. Kymriah^®^, the first CAR-T cell therapy approved by the FDA, has demonstrated effective clinical therapeutic outcomes for the treatment of refractory or recrudescent B-cell precursor acute lymphoblastic leukemia 49, 50. Despite the tremendous clinical achievements of cancer immunotherapy, several significant concerns still remain, which are associated with adverse effects, off-target effects and limited efficacy 51-53.

**Figure 1:**
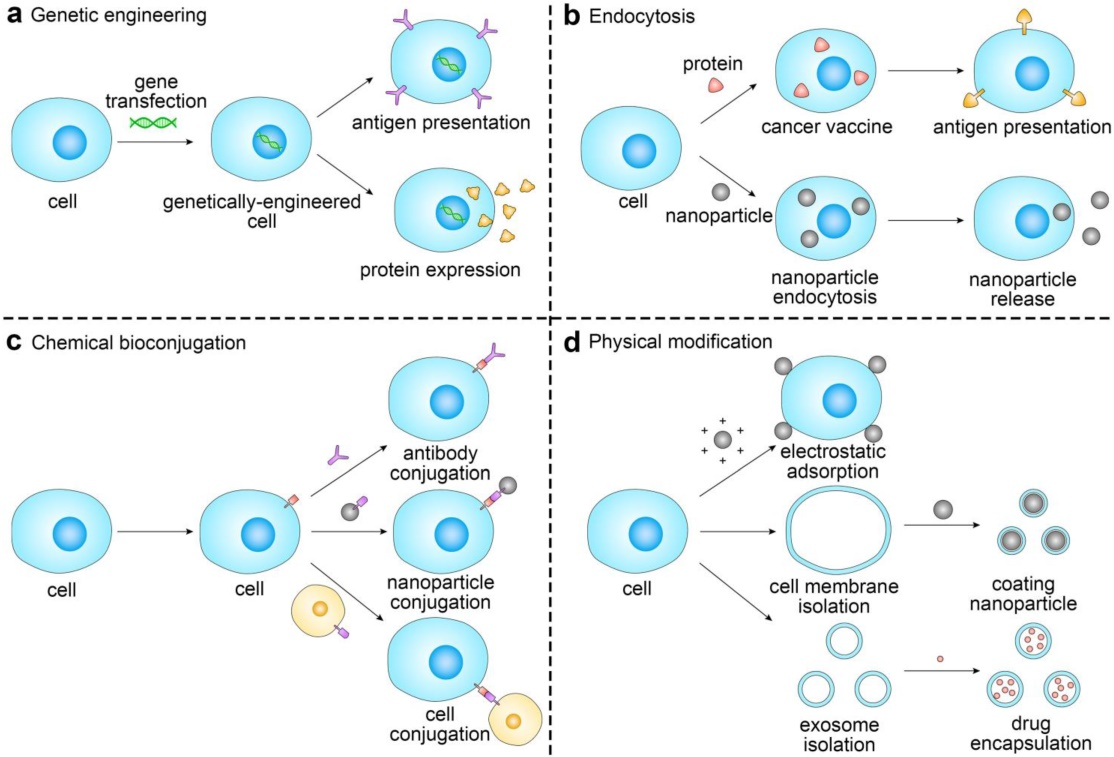
Schematic of the representative strategies of engineering cells for cancer immunotherapy. The representative cells used for drug delivery and cancer immunotherapy involve erythrocytes, platelets, leukocytes, cancer cells and stem cells.

To this end, many novel strategies from the perspectives of drug discovery and drug delivery have been developed. Among them, cell-based drug delivery systems provide a promising platform to enhance delivery efficiency, increase therapeutic efficacy, and reduce off-target and side effects of cancer immunotherapy. By utilizing recent advances in immunotechnology, micro/nanotechnology and molecular pharmaceutics, such cellular systems range from biologically, chemically, and physically-engineered cells to bioinspired and biomimetic nanomedicines (**Figure 1**) 54-56. In this review article, we will focus on recent progress in the field of cell engineering for cancer immunotherapy, and discuss potential future directions of cell engineering approaches for delivery of cancer immunotherapies.

## Engineering cells *via* genetic modification

Genetic engineering aims to change cell phenotypes by altering genetic information 57. A variety of immune cells can be genetically engineered for cancer immunotherapy, including macrophages, natural killer (NK) cells and T cells 58-60. Among them, genetically-engineered T cells have been extensively studied. T cells can be isolated from the peripheral blood or tumor tissue of patients 61. After screening and gene transfection, functionalized T cells are re-administered into the patients to eradicate cancer cells. TCR-T and CAR-T cell therapies are two emerging ACT therapies in which the genetically-engineered cells have preferable targeting capabilities and clinical therapeutic response 5, 62, 63.

TCRs are a characteristic biomolecule of T cells, and consist of *α*- and *β*-chains associated with the CD3 complex composed of *γ*-, *δ*-, *ε*- and *ζ*-chains 61. TCRs are membrane proteins responsible for recognizing specific antigens and mediating intracellular signaling pathways for activation of T cells. This process is mediated by MHCs, kind of polymorphic molecules that are expressed on the APC surface associated with antigens. Interactions between antigens and TCRs result in phosphorylation of immunoreceptor tyrosine-based activation motifs (ITAMs), and therefore activate intracellular signaling in the T cells and release of cytokines, such as interferon-*γ* (IFN-*γ*)/interleukin-2 (IL-2) and cytotoxic proteins, such as perforin/granzyme 61, 64, 65. There are many cancer-associated antigens, which include but are not limited to carcinoembryonic antigen (CEA), B-lymphocyte antigen, glycoprotein 100 (gp 100) and human epidermal growth factor receptor-2 (HER-2) 66-68. However, evidence has shown that cancer cells share similar surface antigens with normal cells, which limits the ability of autologous T cells to distinguish between cancer cells that escape immune eradication and normal cells 69. The TCR-T-based technique is considered to be a promising strategy to decrease cancer cell immune escape by genetically modifying T cells to express receptors with high affinity to the antigens 70. In this strategy, TCR genes derived from tumor-specific T cells or screened by bacteriophage libraries of antibodies are further optimized by substitution of nucleotides to elevate the TCR affinity to the tumor-associated antigens. This affinity-enhanced TCR approach reinforces intracellular signal transduction and therefore enables T cells with augmented activity to kill the cancer cells 61.

TCR-T therapy is often utilized as a therapy for hematological malignancies 71, 72. For example, Tawara *et al.* developed TCR-T cells capable of specifically binding to Wilms tumor 1 (WT1) peptide, a specific epitope on leukemic cells of acute leukemia and myelodysplastic syndrome 73. The engineered TCR-T cells were able to maintain* ex vivo* peptide-specific immune reactivity in the peripheral blood of patients. Hematopoietic function recovery was observed in 40% of patients after treatment. Additionally, TCR-T therapy can also be used for treatment of solid tumors such as melanoma 74, multiple myeloma 75, colorectal 76 and synovial sarcoma 77. Orlando *et al.* identified that the tumor-associated antigen, preferentially expressed antigen in melanoma (PRAME) was a specific epitope on medulloblastoma cells correlated with poor overall survival 78. Enhanced *in vitro* and *in vivo* anticancer activities were observed after treatment with the PRAME-specific TCR-T cells. Meanwhile, lower toxicity of these TCR-T cells introduced with an inducible caspase 9 gene was observed compared with the untransduced control T cells 79.

Recently, two FDA-approved CAR-T cell-based therapies, Kymriah and Yescarta are being utilized for the treatment of patients with acute lymphoblastic leukemia and non-Hodgkin lymphoma, respectively 80, 81. The basic structure of CAR includes antigen-binding, transmembrane and intracellular signaling domains. The antigen-binding domain is a single-chain variable fragment (scFv) derived from the B cell. Since recognition by CAR is MHC-independent, scFv has been widely used regardless of the type of human leukocyte antigen (HLA). CARs recognize antigens on cancer cell membranes, such as CEA, CD19 and vascular endothelial growth factor receptor 2 (VEGFR2), leading to recruitment of signal-initiating molecules, phosphorylation of signaling domains and activation of kinase cascades 82, 83. In design of CAR, the signal-initiating molecules contain the *ζ*-chain of the CD3 complex and the *γ*-chain of the high-affinity receptor for immunoglobulin E (FcεRI) 61. Identification of antigen epitopes on cancer cells is important for CAR design. CD19 on B cell malignancies is an ideal target for CAR. It has been reported that 50-90% of patients respond to anti‑CD19 CAR-T cell therapy 84. However, serious side effects including cytokine-release syndrome and neurotoxicity, which are potentially life-threatening in severe cases are frequently concomitant, which greatly hinders its widespread application in clinic 85. The second and third generations of CARs have been developed for enhanced in vivo persistence and function of CAR-T cells and reduced side effects. The costimulatory molecule genes are transduced into the T cells simultaneously. The expressed CARs include costimulatory signaling domains as a part of the intracellular domain 86. Ying *et al.* constructed an anti-CD19 CAR molecule (CD19-BBz(86)) with intracellular 4-1BB co-stimulatory and CD3ζ signaling domains 87. The CD19-BBz(86) CAR-T cells were safer and more effective than the counterparts without the costimulatory signaling domain owing to release of fewer cytokines and more anti-apoptotic molecules. Six of eleven patients with B cell lymphoma receiving the treatment of CD19-BBz(86) CAR-T cells presented complete remission but no significant increase of cytokine serum level or neurotoxicity.

Efficient activation and expansion of T cells is of the essence in enhancing immunotherapy. The use of commercial expansion beads (Dynabeads) for *ex vivo* expansion of T cells is limited by low efficiency and limited functionality of the T cell products. Cheung *et al.* developed APC-mimic scaffolds (APC-ms) composed of lipid membrane-coated mesoporous silica micro-rods 88. By encapsulation of IL-2 and bioconjugation of anti-CD3 and anti-CD28 antibodies, APC-ms presented superior effects on polyclonal expansion of primary mouse and human T cells than Dynabeads. Elevation of antigen-specific expansion of cytotoxic T cells was achieved by a single simulation using APC-ms compared with the monocyte-derived DCs. Moreover, APC-ms exhibited favorable expansion ability on the restimulated CAR-T cells than Dynabeads, and comparable antitumor efficacy *in vivo*. Due to costly and time-consuming processes of *ex vivo* preparation of CAR-T cells, *in situ* programming of T cells with nanoparticles was proposed by Smith *et al.* 89*.* The CAR gene-encoded plasmid DNA was mixed with a cationic polymer to form nanosized complexes, followed by modification with the T-cell-targeting anti-CD3e f(ab')2 fragments that mediate endocytosis by lymphocytes. When administered to the mice bearing B-cell acute lymphoblastic leukemia, the nanoparticles programmed the circulating T cells and induced tumor regression equivalent to the traditional CAR-T cell therapy.

**Figure 2:**
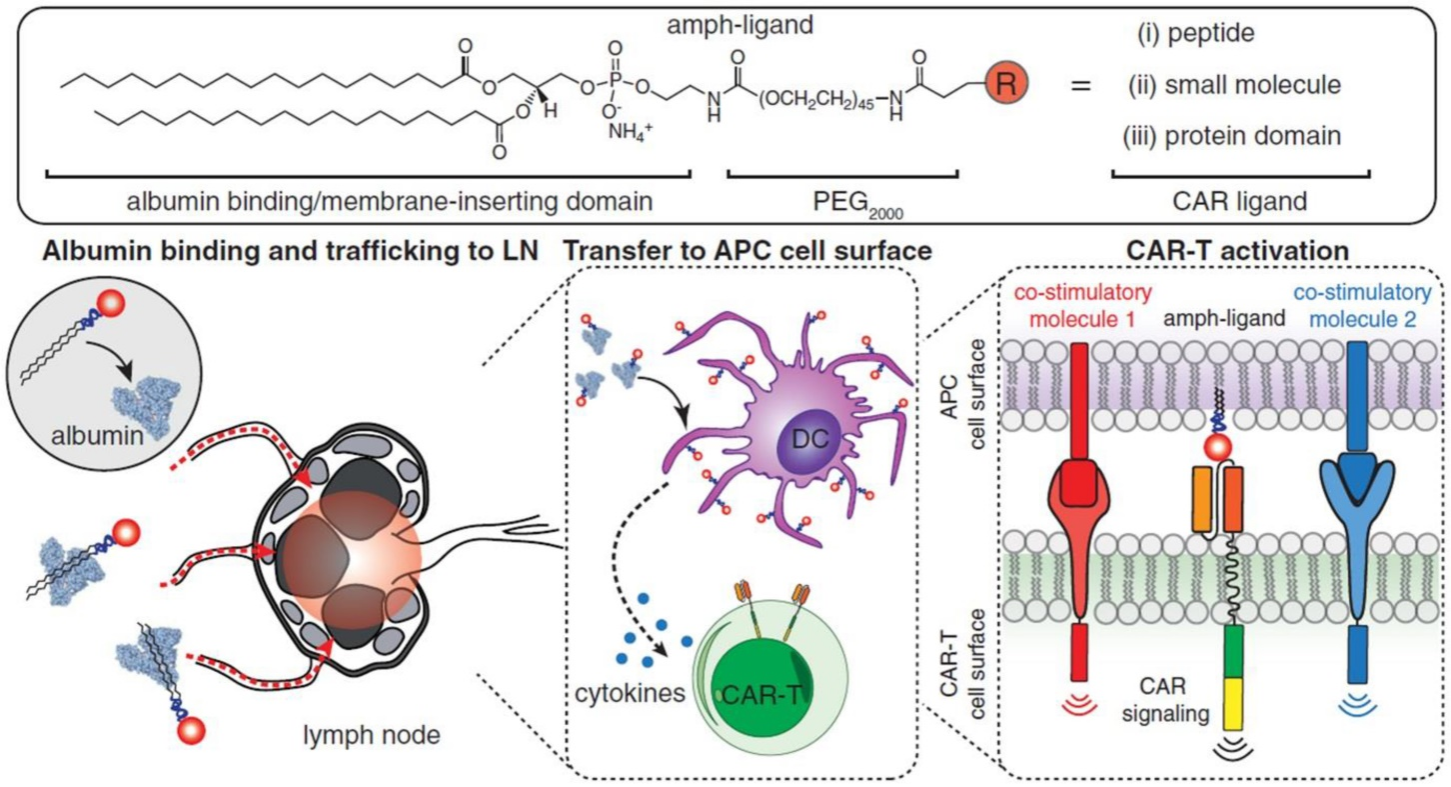
Schematic of the structure of amph-ligands and the process of amph-ligand vaccine-boosting approach. The amph-ligand consists of lipid, PEG and CAR ligand. The long-chain lipid moiety could bind to serum albumin that facilitates the accumulation of the amph-ligand into the lymph nodes. The CAR ligand was subsequently decorated on the surface of DCs, which activated the CAR signaling to increase the expansion of the CAR-T cells. Reprinted with permission from ref 90. **Copyright** 2019 The American Association for the Advancement of Science.

**Figure 3:**
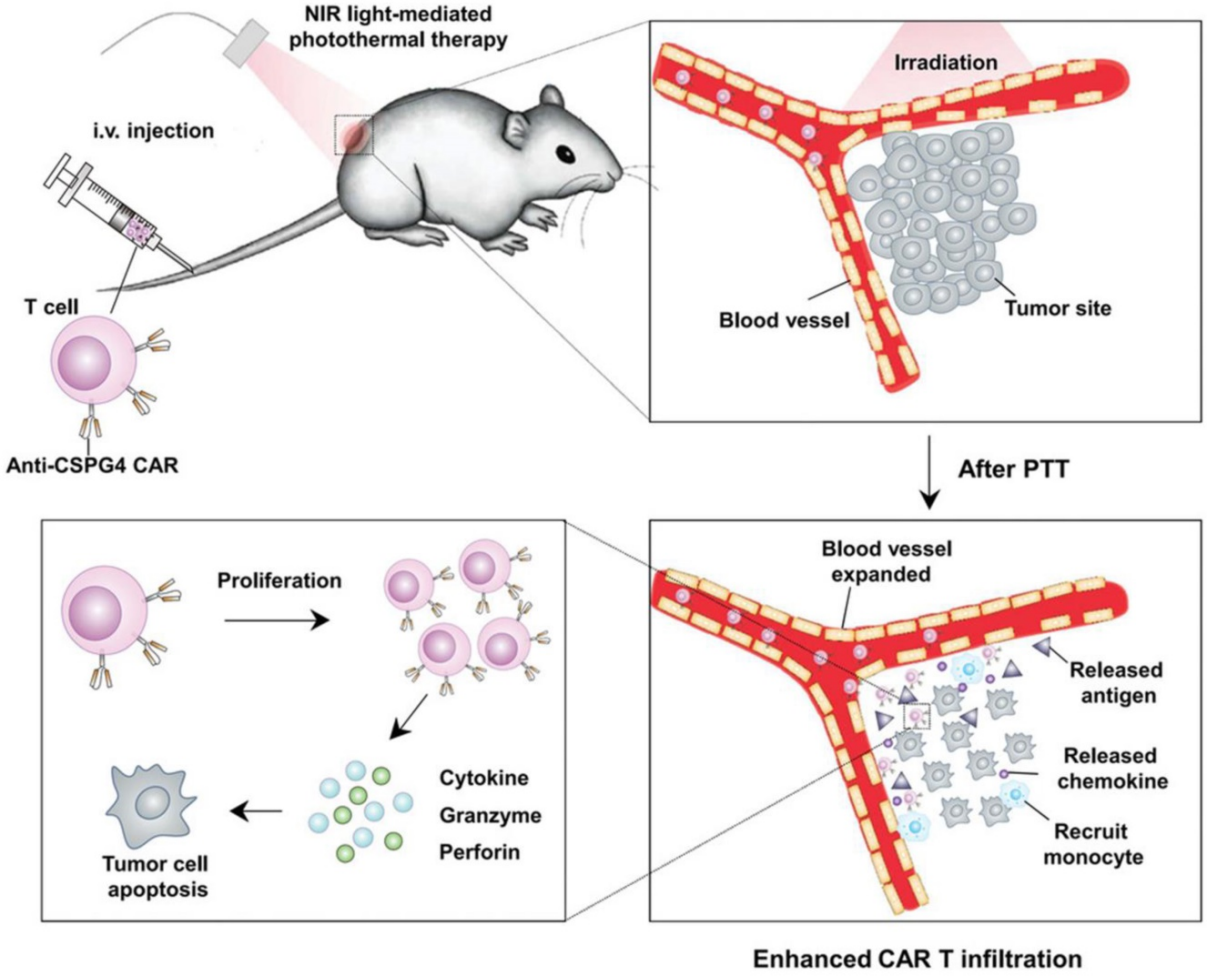
Schematic of enhanced infiltration and activation of the CAR-T cells by mild heating of the tumor. ICG-PLGA nanoparticles were intratumorally injected followed by light irradiation to generate mild heat to disrupt the ECM, which led to reduced interstitial fluid pressure and increased blood perfusion, and therefore enhanced infiltration of the circulating CAT-T cells in the tumor tissue. Reprinted with permission from ref 96. **Copyright** 2019 WILEY-VCH Verlag GmbH & Co. KGaA, Weinheim.

Although effective in treating hematological malignancies, the utilization of CAR-T cells for the treatment of solid tumors is more challenging, which is due in part to limited expansion, poor penetration, and decreased viability of administered CAR-T cells. Recently, Ma *et al.* developed lymph node-targeted amphiphile CAR-T cell ligands (amph-ligands) to directly promote donor cells *via* their chimeric receptor *in vivo* for enhanced efficacy of the CAR-T cell therapy against solid tumors (**Figure 2**) 90. Amph-ligands were composed of phospholipid, polyethylene glycol (PEG) and CAR ligand moieties. After injection, the long-chain alkane of the phospholipid moiety readily bound to the albumin in the blood, which mediated the transport of amph-ligands to the lymph nodes. The CAR ligands were further decorated on the APC surfaces, which primed the circulating CAR-T cells in the lymph nodes. This approach showed its potential to increase the CAR-T cell expansion and augment the antitumor immunity in multiple mouse solid tumor models. On the other hand, IL-7 and CCL19 are regarded to be crucial for the maintenance of the T cell zone in lymphoid organs where DCs and T cells are recruited from the periphery 91, 92. IL-7 enhances proliferation and survival of T cells, while CCL19 is a chemotactic factor for DCs and T cells 93, 94. Adachi *et al*. developed CAR-T cells expressing both IL-7 and CCL19, which could significantly augment the DC and T cell infiltration into the solid tumor compared with the traditional counterpart without cytokine expression 95. To enhance penetration of CAR-T cells into solid tumors, Chen *et al.* applied photothermal pre-treatment to disrupt extracellular matrix (ECM) for enhanced tumor penetration of CAR-T cells (**Figure 3**) 96. Indocyanine green (ICG), a photothermal agent, was loaded into poly(lactic-*co*-glycolic) acid (PLGA) nanoparticles, which were intratumorally injected into the tumor tissue. Upon light irradiation, mild heating generated by the ICG-loaded nanoparticles resulted in the disruption of the ECM followed by decreased interstitial fluid pressure and increased blood perfusion. This photothermal pre-treatment significantly improved the tumor penetration of subsequent intravenously-injected CAR-T cells, leading to enhanced antitumor efficacy in the solid tumors. Cho *et al.* reported that addition of a pair of leucine zippers between the scFv and the intracellular domain controlled the recognition of different antigens by T cells by altering the structure of the leucine zipper-scFv, thereby increasing the functionality of T cells 97. Specifically controlling the type and level of immune response could also be achieved by customized sculpt immune cell response to overcome tumor immunosuppression 98.

In addition to T and B cells, NK cells are another type of lymphocyte, which are critical to the innate immune system and defend the human body against cancer. By secreting cytokines, NK cells regulate immune response and promote maturation of APCs 99, 100. NK cells can also induce the polarization of macrophages to M1 type 101, 102 and target tumor tissue *via* membrane protein, such as natural killer group 2 member D (NKG2D) receptor or DNAX accessory molecule-1 (DNAM-1) 103, 104. Furthermore, NK cells have been reprogrammed with CAR to strengthen recognition specificity and reactivity to cancer cells 105. Jiang *et al.* genetically modified NK-92MI cells with a CAR containing anti-CD138 fragment, which showed significantly enhanced cytotoxicity toward the CD138-positive multiple myeloma cells compared with the CD138-negative counterparts 106. Chu *et al.* developed CS1-specific CAR-expressing NK cells for immunotherapy of multiple myeloma 107. The engineered NK cells displayed specific recognition of multiple myeloma cells overexpressing the CS1 surface protein, which efficiently slowed growth of human multiple myeloma and prolonged mouse survival in the tumor mouse model.

**Figure 4:**
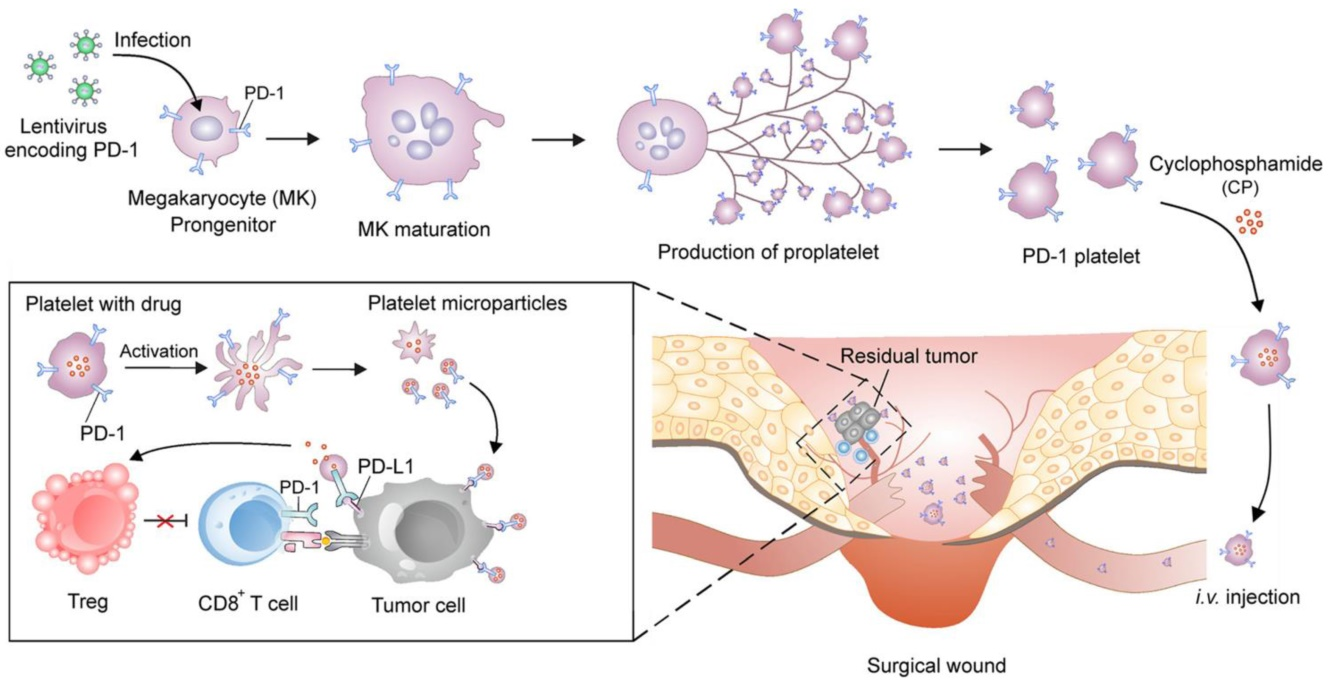
Schematic of the development of the platelets with PD1 presentation and reactivation of the CD8^+^ T cells. The PD1-expressing platelets were obtained from the MKs that were genetically transfected with the lentivirus encoding PD1, and further loaded with CP, an immunosuppressant. The PD1-expressing platelets with CP could efficiently accumulate to the surgical wounds and release both PD1 and CP. The released PD1 blocked the PDL1 on the tumor cells to reactivate the CD8^+^ T cells, while the released CP depleted the Tregs to enhance the activity of the CD8^+^ T cells. Reprinted with permission from ref 108. **Copyright** 2018 American Chemical Society.

Zhang *et al.* engineered platelets decorated with PD1 for preventing post-surgical cancer recurrence (**Figure 4**) 108. Megakaryocytes (MKs) that are responsible for *in vitro* large-scale production of platelets were genetically modified using lentivirus encoding PD1. The obtained PD1-presenting MKs was able to generate mature platelets with PD1, which increased the accumulation of PD1 to surgical wounds by relying on the physiological function of platelets as monitors of vascular injury. PD1 was released *via* platelet-derived microparticles from cell membranes upon platelet activation and blocked PDL1 on tumor cells to reinvigorate the exhausted CD8^+^ T lymphocytes. Cyclophosphamide (CP), an immunosuppressant, was simultaneously delivered by the PD1-expressing platelets to exhaust the regulatory T cells (Tregs) and enhance the cytotoxic effects of the CD8^+^ T cells. Xue *et al.* conjugated granulocyte macrophage-colony stimulating factor (GM-CSF) mRNAs to polypeptide linker covalently to construct fusion gene GC2A, which was further inserted into the adenovirus vector to transfect human embryonic kidney 293T (HEK293T) cells 109. The recombinant adenovirus proliferated in HEK293T cells and released GM-SCF that promoted the DC proliferation and differentiation in the tumor-bearing mice.

## Engineering cells *via* endocytosis-mediated functionalization

Endocytosis is an approach for cell engineering in which autologous cells are treated with proteins, drugs or nanoparticles *in vitro*, followed by reinfusion to the body. Depending on the phagocytosed substances, the representative approaches include vaccine endocytosis for antigen presentation and nanoparticle endocytosis for drug delivery 110-112.

**Figure 5:**
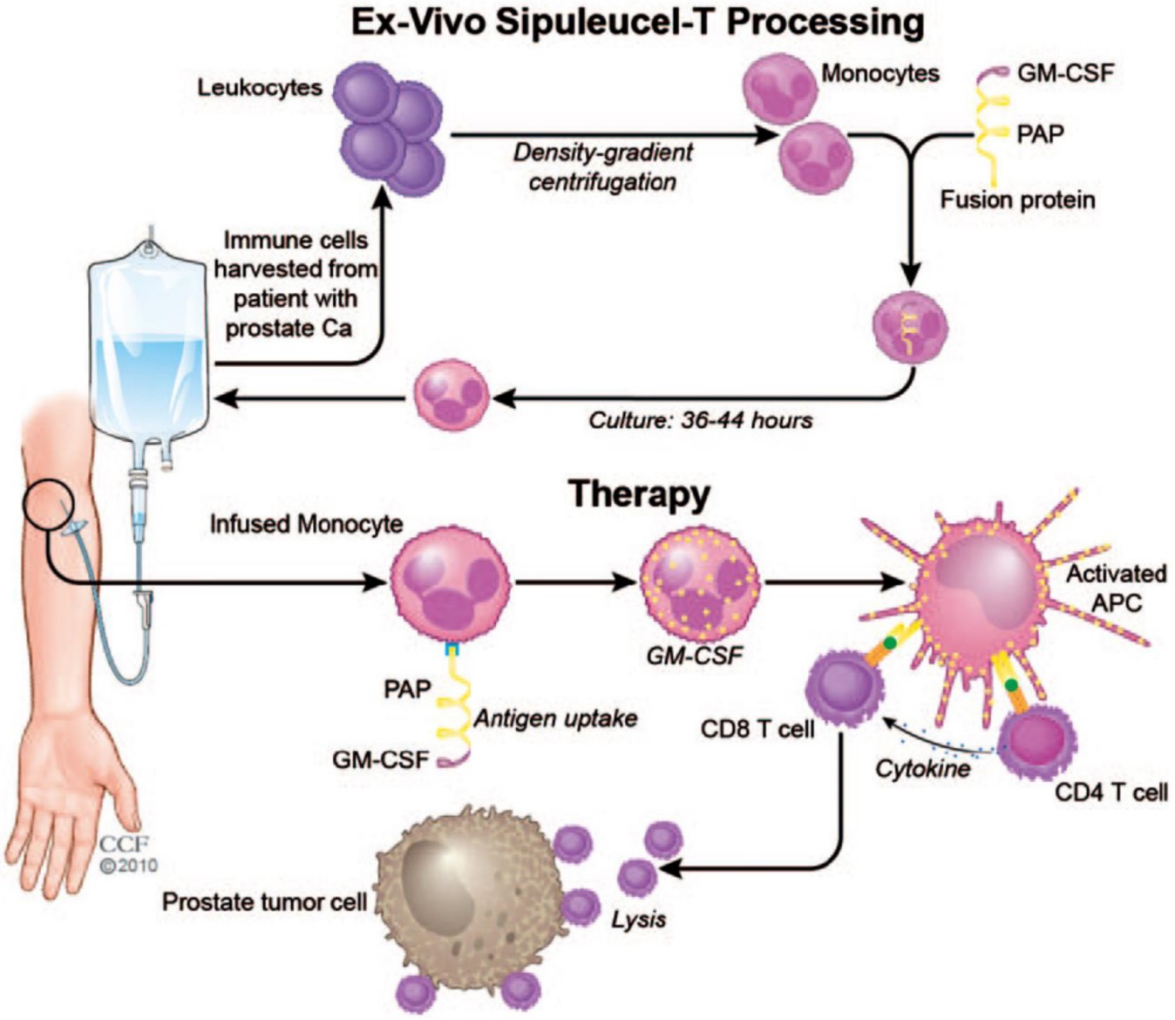
Schematic of the process of the Sipuleucel-T therapy against prostate cancer. The monocytes were harvested from the patients and treated with the fusion protein, and re-infused into the patients. The infused monocytes could differentiate to the APCs, which activated the PAP-specific CD4^+^ and CD8^+^ T cells to kill the prostate cancer cells. Reprinted with permission from ref 113. **Copyright** 2011 SAGE Publications.

Research of cancer vaccines reached a milestone with the development of Sipuleucel-T for treating patients with metastatic castration-resistant prostate cancer in 2010 (**Figure 5**) 113. Leukocytes are harvested from the patients' peripheral blood, and monocytes are isolated by density gradient centrifugation. The harvested monocytes are cultured with fusion protein for 36-44 hours to allow for endocytosis of the fusion protein. The recombinant fusion protein used is PA2024, which is composed of a prostatic acid phosphatase (PAP) domain and a GM-CSF domain. PAP is highly expressed on prostate cancer cell membranes and serves as a target antigen for T cells. GM-CSF enhances proliferation of monocytes and promotes their differentiation to APCs. After administration, engineered monocytes first differentiate into APCs and in succession, activate the PAP-specific CD4^+^ and CD8^+^ T cells. The CD8^+^ T cells induce cell lysis of the prostate tumor cells with the help of cytokines secreted by CD4^+^ T cells. Sipuleucel-T showed prolonged median survival of 4.1 months with mild side effects 30, 113. With the exception of Sipuleucel-T, no other cell-based vaccines have yet passed clinical trials due to limited therapeutic efficacy 114. Accordingly, it remains a challenge to develop cancer vaccines with clinically significant anticancer activity 115-117.

**Figure 6:**
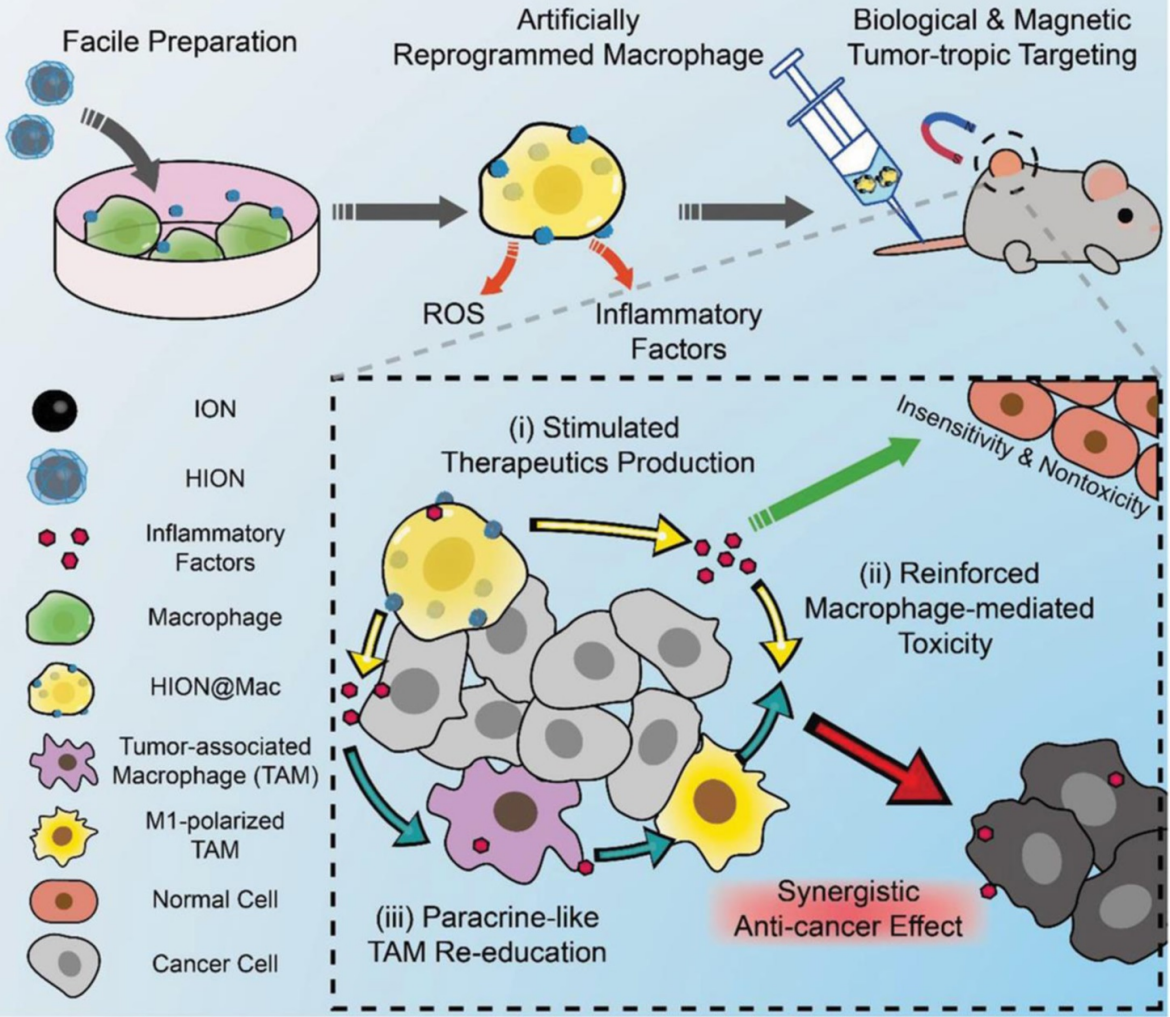
Schematic of the artificially reprogrammed HION@Mac for cancer therapy. Reprinted with permission from ref 138. HION@Mac was obtained by the macrophage internalizing HION, the hyaluronic acid-coated iron oxide nanoparticles. HION@Mac after injection could migrate to the tumor with expression of inflammatory factors *via* chemotaxis of the macrophage. In the tumor tissue, HION@Mac not only induced tumor cell apoptosis by releasing ROS and inflammatory factors, but also facilitated the polarization from the TAMs to the M1-type macrophages for enhanced antitumor efficacy. **Copyright** 2019 WILEY-VCH Verlag GmbH & Co. KGaA, Weinheim.

Like fusion proteins, nanoparticles can also be internalized by cells to construct a cell-based drug delivery system to combine the advantages of nanoparticles and cells for augmented immunotherapeutic efficacy 118. Nanoparticles enhance the accumulation of anticancer drugs in solid tumors through the enhanced penetration and retention (EPR) effect, which is the phenomenon whereby nanoparticles tend to preferentially accumulate in the tumor microenvironment due to its leaky vasculature 119. However, recent studies suggest that the EPR-based passive-targeting effect of nanoparticles is inadequate 120-122. Combination of the distinct advantages of nanoparticles and cells is a promising strategy to achieve enhanced tumor accumulation 123-125. The immunosuppressive microenvironment is one of the primary obstacles to the effectiveness of immunotherapeutics 126,127. A number of immunosuppressive factors have been identified using the molecular imaging techniques 128, 129, and a variety of strategies have been proposed to overcome the immunosuppressive tumor microenvironment in order to activate immune response and improve cancer immunotherapy 130-132. Li *et al.* prepared a dendritic cell-based nanodiamond delivery system 133. The nanodiamond (denoted as Nano-DOX) was covalently conjugated with doxorubicin (DOX), a clinically-used anticancer drug and cyclictripeptides (RGD), a tumor-targeting ligand. Nano-DOX was efficiently internalized by DCs that were isolated from mouse bone marrow to form Nano-DOX-DC. After intravenous injection into athymic mice bearing orthotopic human glioma xenografts, Nano-DOX-DC crossed the blood-brain barrier and entered into the glioma tissue, which was followed by the release of Nano-DOX into the tumor microenvironment. Nano-DOX induced emission of damage associated molecular patterns (DAMPs), including calreticulin, high-mobility group box 1 protein (HMGB1) and adenosine triphosphate, which increased the immunogenicity and antigenicity of the glioma cells and subverted the tumor-associated immunosuppression 134. The enhanced immunogenicity stimulated maturation of DCs, which in turn promoted maturation of the innate and supplementary lymphocytes. Nano-DOX-DC exhibited greater antitumor efficacy compared with the blank DCs without Nano-DOX. The monocytes and macrophages carrying Nano-DOX also showed their preferable antitumor efficacy for glioma treatment 135, 136. Jin *et al.* prepared magnetic nanoparticles to enhance the enrichment of DCs in the lymph nodes. The DCs containing the magnetic nanoparticles could be directed to the lymph node upon the external magnetic field, therefore increasing the lymphatic targeting of DCs and enhancing the anticancer effects 137. Recently, Li *et al.* developed macrophage-based drug delivery systems to reverse the immunosuppressive microenvironment in the tumor tissue (**Figure 6**) 138. Superparamagnetic iron oxide nanoparticles were prepared and further modified with hyaluronic acid, followed by internalization by naive macrophage (designated as HION@Mac). Inflammatory signals drove HION@Mac to accumulate in tumor tissue where HION@Mac secreted reactive oxygen species (ROS) and inflammatory factors to induce apoptosis of tumor cells. HION@Mac also demonstrated its potential to polarize intratumoral tumor-associated microphages (TAMs) to M1-type macrophages. These two pathways jointly contributed to immune activation and tumor cell apoptosis, resulting in a synergistic anticancer effect.

## Engineering cells *via* chemical bioconjugation

Endocytosis may involve concerns about the instability of nanoparticles and unexpected release of drugs by endocytic vesicle-mediated degradation 139. Bioconjugation of cargoes on cell membranes is an alternative approach 140. To improve the tumor-targeting efficiency of anti-PDL1 and reduce its off-target effects, Wang *et al.* developed platelets conjugated with anti-PDL1 (P-aPDL1) that could travel to the surgical site in order to inhibit post-surgical tumor recurrence 141. P-aPDL1 was obtained by conjugating aPDL1 to the surface of platelets using a SMCC crosslinker containing amine- and sulfhydryl-reactive groups. aPDL1 was stably bound to the non-activated platelets, which facilitates delivery of aPDL1 to the residual tumors at the surgical site. Upon activation of platelets, expression of PDL1 in the tumor tissue was upregulated, and aPDL1 was released due to generation of platelet-derived microparticles from cell membranes of platelets. The released aPDL1 blocked PDL1 on the tumor and antigen-presenting cells. P-aPDL1 was demonstrated to enable controlled delivery of PDL1 and potent recurrence inhibition on the post-surgical mouse models with melanoma and triple-negative breast carcinoma (TNBC). Han *et al.* applied P-aPDL1 to inhibit tumor relapse and metastasis after thermal ablation 142. Photothermal therapy is limited because remnants of microtumors are often responsible for local recurrence and distant metastasis. P-aPDL1 therapy exhibited efficient targeting capacity to an incompletely ablated tumor based on damaged vascular microenvironment after photothermal treatment and therefore produced significantly augmented therapeutic efficacy on the xenograft breast tumor mouse model. Such platelet-mediated drug delivery system could also effectively target the residual microtumors after treatment with high-intensity-focused-ultrasound ablation.

**Figure 7:**
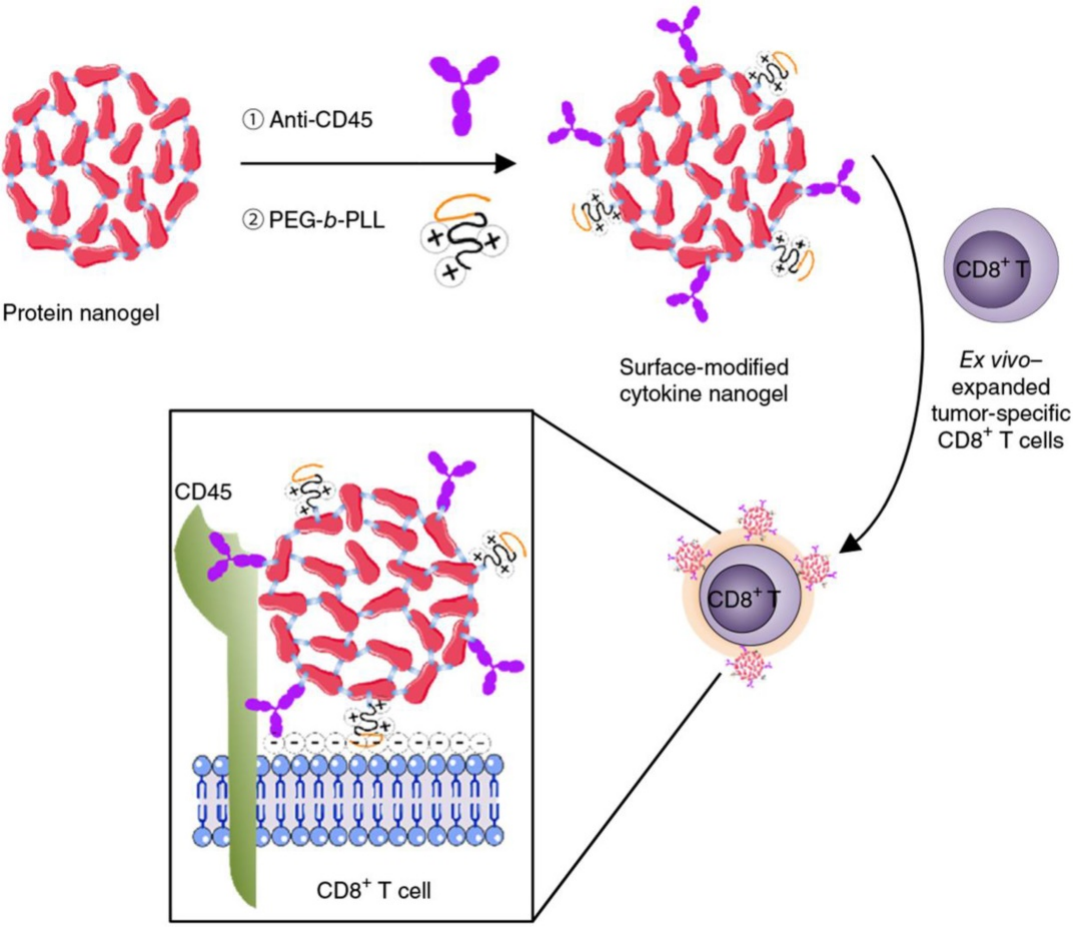
Schematic of the modification of cytokine nanogel on the T cell surface for cancer immunotherapy. IL-15 was self-crosslinked to prepare the IL-15 nanogel, which was subsequently modified with the anti-CD45 antibodies and the cationic PEG-b-PLL polymer. The surface-modified IL-15 nanogels could be stably attached on the surface of T cells *via* a combination of electronic and antibody-receptor interactions. The obtained T cell backpack showed superior effects in augmenting immunotherapeutic efficacy. Reprinted with permission from ref 146. **Copyright** 2018 Springer Nature.

Cellular backpacks using nanoparticles without being internalized also hold promise of targeted drug delivery and enhanced cancer immunotherapy, which integrates the respective merits of nanoparticles as drug depots and cells with natural directional migration potency as active carriage 143, 144. Huang *et al.* reported nanoparticle-conjugated T cells for enhanced targeting of chemotherapeutics to disseminated tumor cells in lymph nodes 145. Topoisomerase I inhibitor, SN-38 was encapsulated in lipid nanoparticles decorated with maleimide headgroups, which were linked on the T cell surface with a high level of reduced thiol groups by maleimide-thiol coupling. The engineered T cells showed superior lymph-targeting capacity, which rendered the quantity of SN-38 in lymph nodes 90-fold higher than that of free drug that was intravenously injected at 10-fold higher dosage. Tumor burden was significantly reduced and survival period was markedly prolonged by the SN-38-loaded nanoparticle-functionalized T cells in comparison with either free SN-38 or SN-38-loaded nanoparticles. Tang *et al.* developed a cytokine nanogel-decorated T cell-mediated delivery system for enhanced T cell function and cancer immunotherapy (**Figure 7**) 146. Repetitive units of interleukin-15 (IL-15) were crosslinked with themselves by a synthetic disulfide-containing bis-*N*-hydroxy succinimide crosslinker to form a “carrier-free” protein nanogel, which was later decorated with poly(ethylene glycol)-*co*-poly(lysine) (PEG-*b*-PLL) and anti-CD45 antibodies. The cationic PEG-*b*-PLL polymer facilitated electrostatic absorption of nanogels onto T cells, while anti-CD45 antibody increased cell surface retention of nanogels by preventing internalization. Elevation of surface reduction potential of T cells following antigen recognition in lymph node and tumor tissues led to nanogel collapse and IL-15 release. This controlled manner brought about an 8-fold higher maximum tolerated dosage of IL-15 and a 16-fold amplification of T cells. The mouse T cells and human CAR-T cells backpacking the nanogels with large quantities of IL-15 presented superior antitumor effects in mouse melanoma and glioblastoma models. Apart from IL-15, similar strategy was adopted for delivery of IL-2 to overcome the IL-2-induced vascular leak syndrome. A sustained and slow release of IL-2 was achieved by the IL-2 nanogel backpacks, leading to more CD8^+^ memory precursor differentiation and less T-cell exhaustion compared with free IL-2 147.

**Figure 8:**
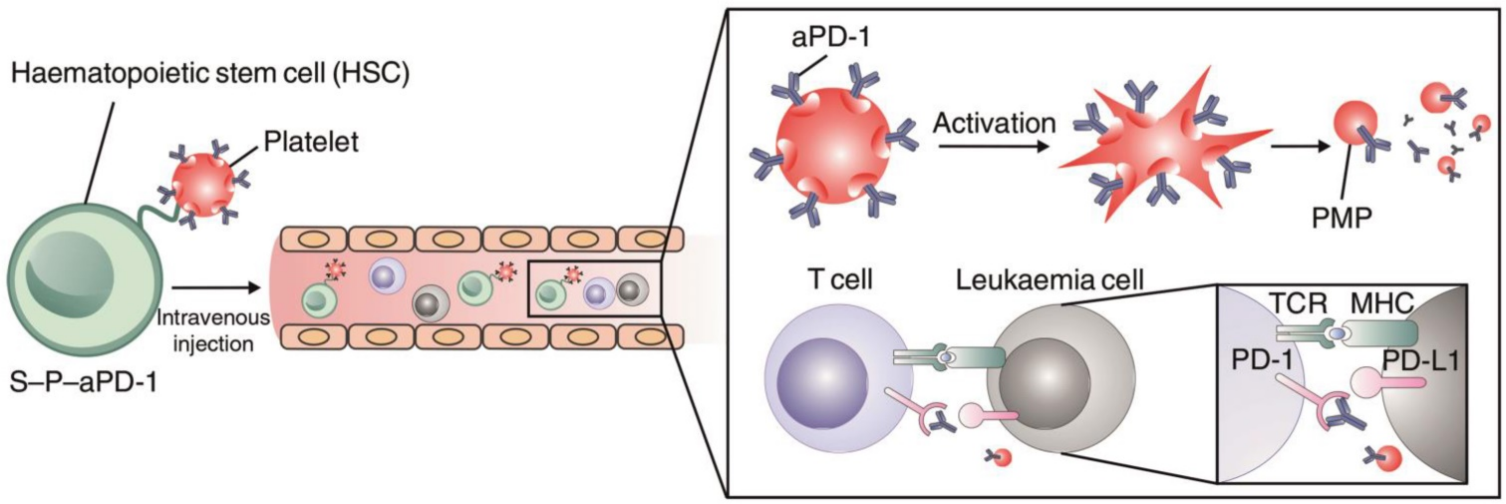
Schematic of the HSC-platelet assembly-assisted delivery of aPD1. The S-P-PD1 elevated the distribution of aPD1 in the leukemia site by the targeting ability of HSCs, thereby yielding enhanced anticancer effects in suppressing the leukemia proliferation and recurrence. Reprinted with permission from ref 148. **Copyright** 2018 Springer Nature.

In addition to modification with antibodies or nanoparticles for cell engineering, Hu *et al.* conjugated anti-PD1 antibody (aPD1)-decorated platelets to hematopoietic stem cells (HSCs) to enhance the delivery of aPD1 to the leukemia site in order to inhibit leukemia growth and relapse (**Figure 8**) 148. The HSC-platelet-aPD1 conjugate (S-P-PD1) could efficiently migrate to bone marrow after intravenous administration into leukemia-bearing mice and release aPD1 locally at the leukemia site upon platelet activation. Treatment with S-P-PD1 resulted in evidently enhanced anti-leukemia effects and prolonged survival of the mice through elevation of the ratio of active T cells and generation of cytokines. This “cell combination” delivery strategy displayed potent effects in improving the anticancer activity of checkpoint blockade therapy by combining the leukemia-targeting capabilities of HSCs and the controlled drug delivery property of platelets.

## Engineering cells *via* physical modification

**Figure 9:**
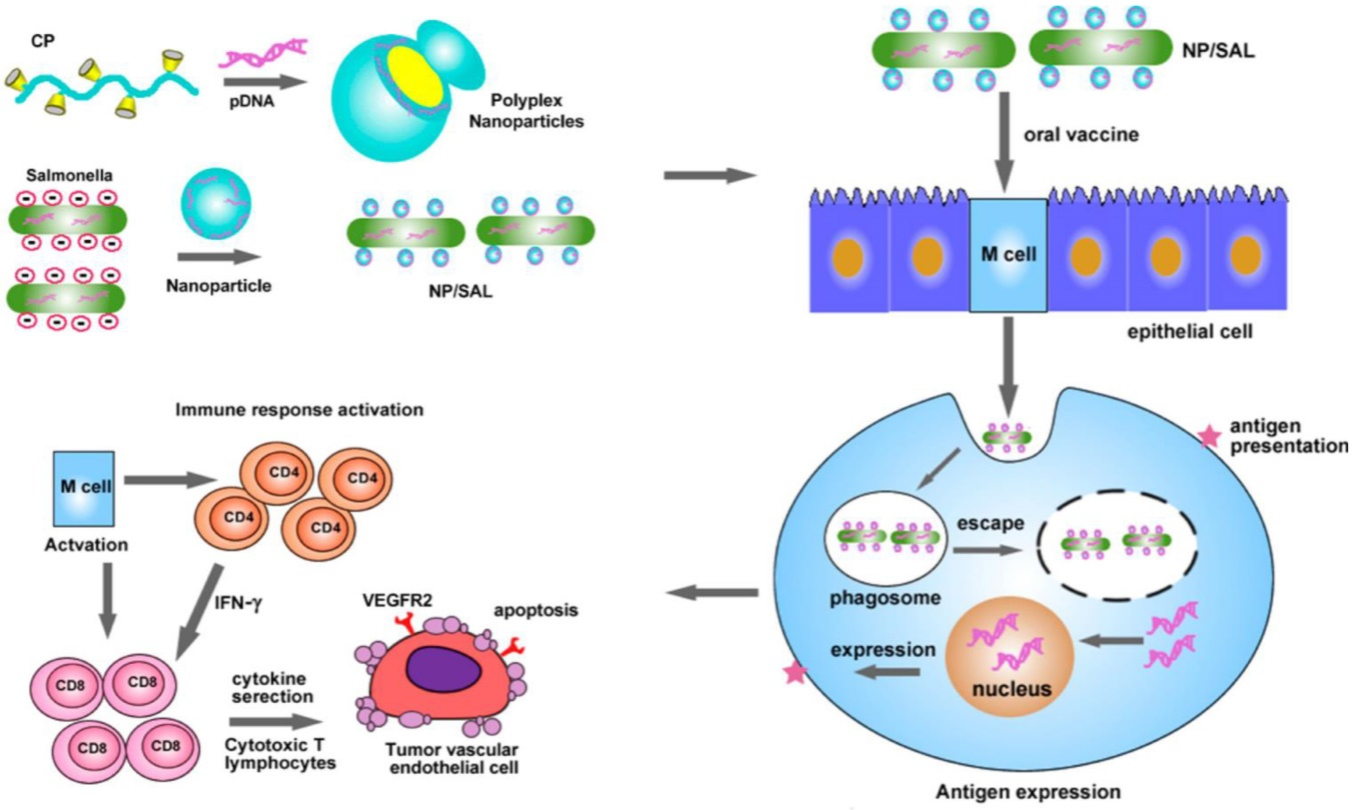
Schematic of oral delivery of the cationic nanoparticle-coated attenuated *Salmonellae* for cancer immunotherapy. The cationic nanoparticles loaded with the DNA plasmid encoding VEGFR2 were coated on the surface of the attenuated *Salmonella*. After oral administration, the bacteria delivered the VEGFR2 gene into the M cells, which expressed the VEGFR2 antigen to activate the CD4^+^ and CD8^+^ T cells. The activated CD8^+^ T cells eliminated the VEGFR2-expressing tumor vascular endothelial cells to inhibit the tumor angiogenesis and growth. Reprinted with permission from ref 149. **Copyright** 2015 American Chemical Society.

Non-covalent physical modification is a convenient strategy for engineering cells, often enabling high activity of cargoes linked to the cellular carriers compared to covalent bioconjugation. Hu *et al.* used cationic polymers to condense the DNA plasmid encoding VEGFR2 and *Salmonella*, a low-cost live attenuated bacteria as a carrier for oral delivery of DNA-based vaccines (**Figure 9**) 149. Electrostatic interaction between cationic polymer and anionic DNA promoted formation of nanoparticles whose surface potential could be adjusted by the ratio of polymer and DNA. The bacteria-shuttled vaccine had excellent stability and could survive under gastric acidity after oral administration. With the aid of cationic nanoparticles, the vaccine can be efficiently taken up by the M cells that serve as protectors for antigen internalization and transportation in the intestine. VEGFR2, which was efficiently expressed by the M cells, acted as an antigen to activate the CD4^+^ and CD8^+^ T cells to eradicate the VEGFR2-expressing tumor vascular endothelial cells. This bacteria-mediated DNA-based vaccine revealed higher effect in inhibiting tumor growth due to increased angiogenesis suppression and tumor necrosis.

**Figure 10:**
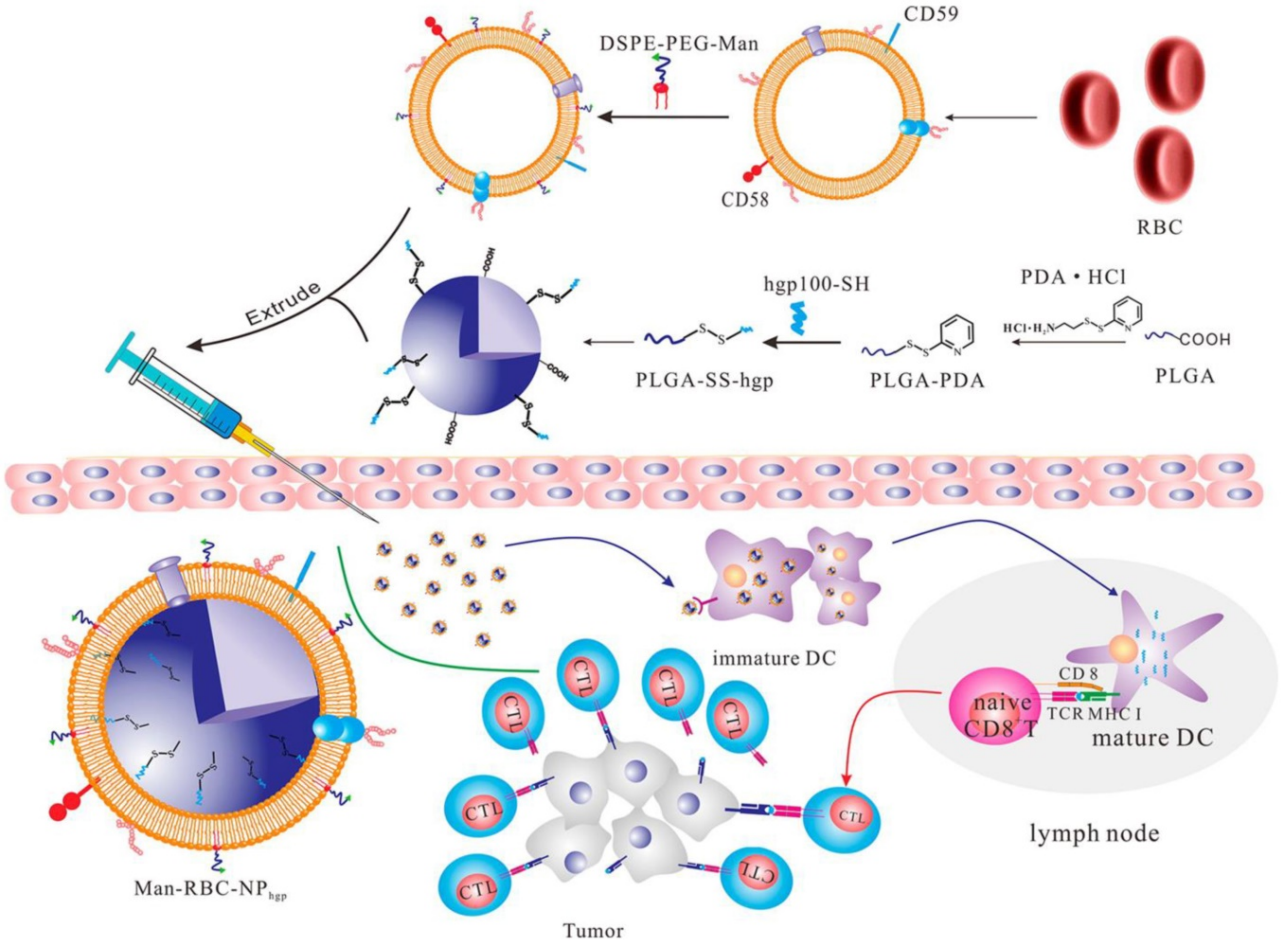
Schematic of the RBC membrane-coated PLGA nanoparticles for production of antitumor immunity. The hgp100-conjugated PLGA nanoparticles were coated with the RBC membranes that were modified with the DSPE-PEG-mannose conjugate. The subcutaneously-injected nanoparticles were taken up by the immature DCs with expression of mannose receptors, and released hgp100 to activate the DCs and subsequently the CTLs to kill the cancer cells. Reprinted with permission from ref 158. **Copyright** 2015 American Chemical Society.

**Figure 11:**
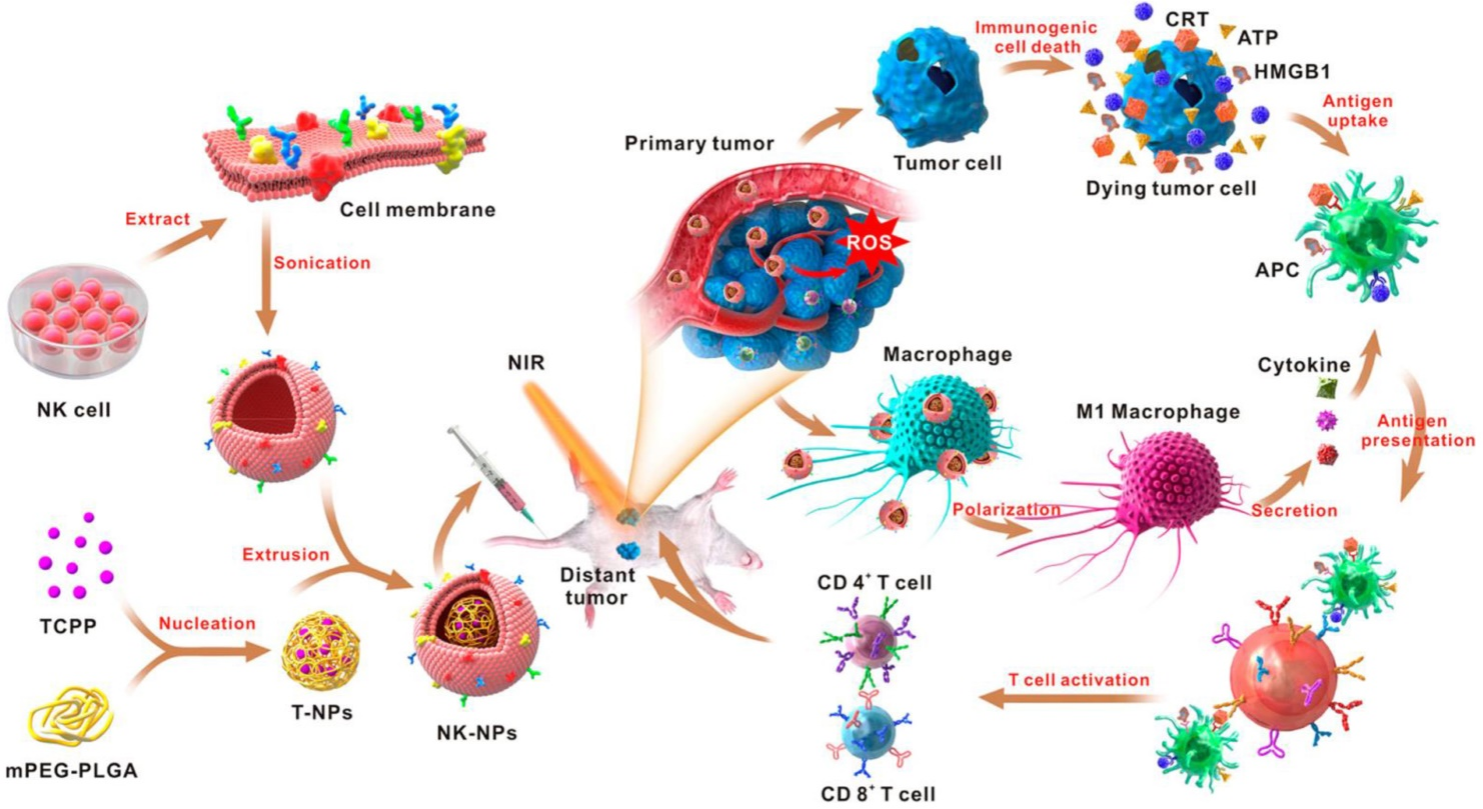
Schematic of the NK cell-membrane-cloaked nanoparticles for augmented immunotherapy based on photodynamic therapy. The TCPP-loaded NK-cell-membrane-coated nanoparticles increased the intratumoral accumulation of TCPP, which produced ROS upon light irradiation to induce tumor cell apoptosis. The proteins expressed on the NK cell membranes could also promote the M1-type macrophage polarization to augment the anticancer immune response. Reprinted with permission from ref 154. **Copyright** 2018 American Chemical Society.

In addition to living cells, cell membranes have been widely used to develop biomimetic drug delivery systems. Preserving cell membrane integrity and a significant portion of membrane proteins facilitates protecting drugs from degradation or activating an immune response 55, 150. Cell membranes from different types of cells have been reported to camouflage nanoparticles, including blood cells 151-153, immune cells 154, 155 and even cancer cells 150, 156. The abundance of red blood cells (RBCs) and their lack of organelles render the RBC membranes favorable for drug delivery applications. The RBC membrane-cloaked nanoparticles revealed longer circulation time and less immunological rejection compared with the free nanoparticles 157. Hu *et al.* prepared nanovaccines with notable antivirulence efficacy by absorbing intact pore-forming toxins on RBC membrane-coated nanoparticles, which laid a foundation for new vaccine design 152. Guo *et al.* reported a RBC membrane-based core-shell drug delivery system for melanoma immunotherapy (**Figure 10**) 158. The nanoparticles prepared by melanoma-associated antigenic peptide hgp100 conjugated PLGA polymers were cloaked by RBC membrane to form a core-shell structure. The RBC membrane was modified with DSPE-PEG-mannose, a kind of polysaccharide that can bind to the mannose receptor on the immune cells such as macrophages and DCs for antigen recognition 159, 160. After administration, the RBC membrane-camouflaged PLGA nanoparticles were easily phagocytosed by immature DCs *via* mannose receptor-mediated interaction, in which hgp100 was released due to the high intracellular level of glutathione 161. Activation of DCs by hgp100 in lymph nodes promoted production of cytotoxic T lymphocytes (CTLs) and led to massive tumor regression. Deng *et al.* prepared NK cell membrane-camouflaged nanoparticles (NK-NPs) by disguising photosensitizer-loaded nanoparticles with the NK cell membranes (**Figure 11**) 154. Once entering circulation system, NK-NPs preferably accumulated in tumor tissue and were engulfed by cancer cells *via* the receptor-mediated interaction. Upon near-infrared (NIR) irradiation, a build-up of ROS generated by tetra(4-carboxyphenyl)porphine (TCPP) as a photosensitizer induced apoptosis of tumor cells. Antigens on dead tumor cells were presented to the T cells by APCs, which activated the T cells to kill remnant tumor cells. On the other hand, proteins on the NK cell membranes stimulated M1-type polarization of macrophages, which further secreted pro-inflammation cytokines to activate APCs for a durable immune response. Xie *et al.* reported cancer cell membrane-coated glucose oxidase-loaded mesoporous silica nanoparticles (MSNs) for cancer immunotherapy 156. Specific antigens expressed by tumor cells activated the immune system, which indicates that coating nanoparticles with cancer cell membranes is a superb approach to elicit an immune response for cancer immunotherapy. Moreover, the homing effect of tumor cells is inherited by the cell membrane, which supports elevated tumor-targeting capabilities. Murine melanoma cells were lysed with good preservation of membrane proteins, including homologous target proteins and immune escape proteins, such as CD47. The cancer membrane-coated MSNs were translocated to the tumor tissue by membrane proteins after intravenous injection. Nutritional supply of tumor cells was cut off by glucose oxidase, which results in cell apoptosis by converting glucose to gluconic acid and hydrogen peroxide. Treatment with the obtained MSNs can efficiently enhance the PD1-based immune checkpoint blockade effect by boosting the production of effector cells *via* DCs, which were activated by the proteins presented on the cancer cell membrane. This starvation strategy combined with immunotherapy resulted in a synergistic anti-tumor effect. Apart from coating nanoparticles with one kind of cell membrane, mixed cell membrane decorated nanoparticles were also investigated to enhance immune response. Liu *et al*. prepared a cytomembrane consisting of fused cells from DCs and cancer cells to coat nanoparticles (NP@FM). NP@FM presented whole tumor antigens from DC membrane and endogenous tumor antigens from cancer cell membrane, and enhanced the activation of T cells 162.

**Figure 12:**
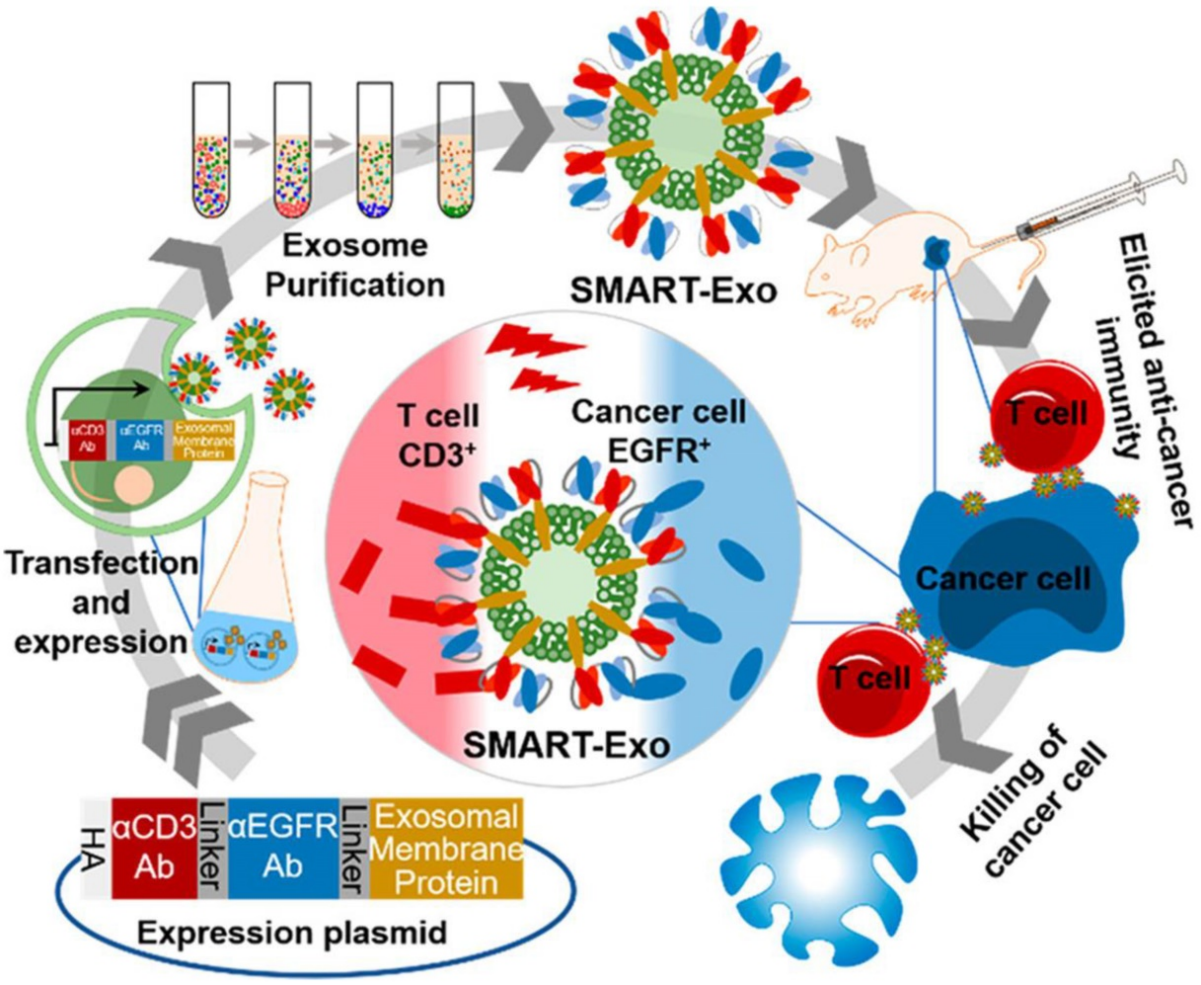
Schematic of the design and production of SMART-Exo for cancer immunotherapy. SMART-Exo with surface expression of both CD3^+^ and EGFR antibodies were obtained from the HEK293 cells transfected with the DNA plasmid encoding the antibodies. SMART-Exo produced the anticancer immunity by inducing the crosslink between the T cells and the EGFR-expressing cancer cells. Reprinted with permission from ref 168. **Copyright** 2018 American Chemical Society.

Exosomes, which are membrane vesicles with a size of 30-100 nm derived from various cell types and resemble cell membranes, are vital for cell communication and take along a collection of biological information from the donor cells 163-165. Thus, exosomes have been increasingly investigated and utilized as drug delivery carriers for cancer immunotherapy 166, 167. Cheng *et al.* transfected HEK293 cells with the DNA plasmid encoding three different proteins: CD3 antibody for T cell recognition, endothelial growth factor receptor (EGFR) antibody for breast cancer cell targeting and membrane protein for surface anchoring (**Figure 12**) 168. The successfully-transfected cells could express all three proteins, and the harvested exosomes (denoted as SMART-Exo) also carried these proteins. SMART-Exo could induce crosslink between the T cells and breast cancer cells, causing the cancer cell apoptosis. *In vitro* cell experiments verified the T cell activation ability of SMART-Exo and *in vivo* studies revealed the engineered exosomes significantly suppressed tumor growth in the xenograft TNBC mouse models. Morishita *et al.* isolated murine melanoma cell-derived exosomes that contain cancer cell antigens and can activate APCs to provoke antitumor immunity 169. The melanoma cells were transfected with plasmid DNA encoding streptavidin-lactadherin protein, a fusion protein anchored on membrane that would further be transferred to exosomes. The biotinylated CpG DNA that encodes proteins facilitates presenting the tumor antigens to DCs was conjugated to the exosome membrane *via* the biotin-streptavidin interaction. The functionalized exosomes exhibited superior antitumor efficacy in tumor-bearing mice in comparison to co-administration of exosomes and CpG DNA.

## Conclusion and Outlook

In order to enhance the efficacy and reduce the adverse effects of cancer immunotherapy, cell-based immunotherapies have attracted considerable attention today, mainly because of their potential biocompatibility and dynamic physiological functions involving immune interactions. In addition to genetically engineered cell therapies such as CAR-T and TCR-T cell therapies, other kinds of cell-based delivery systems have also been utilized for cancer immunotherapy. Augmenting the antitumor immune response and overcoming immunosuppression are the two main goals for cancer immunotherapy. The combination of immunotherapeutic drugs with cell-based delivery systems can potentially enhance the efficiency or efficacy of cancer immunotherapy, mainly reflects in protecting the drug from unexpected degradation, increasing accumulation in target sites, such as tumor and lymph node and/or boosting the immune response and overcoming the tumor immunosuppression to combat with tumor growth, recurrence or metastasis.

Although remarkable advantages have been validated when combining immunotherapy with cells or cell-derived delivery systems, many issues should be taken into account for accelerating clinical translation. For example, preparation processes of these engineered cells or cell-based delivery systems are often complicated 170, 171, causing difficulty in large-scale manufacturing, particularly for the cells that are in small quantity and difficult to harvest, which must be further exploited and optimized for practical application. Moreover, characterization of engineered cells must be comprehensively performed in order to obtain a detailed understanding of the delivery mechanism, which is essential to boost therapeutic efficacy and address safety risks *in vivo*. Strict criteria for quality control should be established in order to achieve reproducible engineering approaches with quality assurance.
